# Interaction of α9α10 Nicotinic Receptors With Peptides and Proteins From Animal Venoms

**DOI:** 10.3389/fncel.2021.765541

**Published:** 2021-12-23

**Authors:** Victor Tsetlin, Yves Haufe, Valentina Safronova, Dmitriy Serov, PranavKumar Shadamarshan, Lina Son, Irina Shelukhina, Denis Kudryavtsev, Elena Kryukova, Igor Kasheverov, Annette Nicke, Yuri Utkin

**Affiliations:** ^1^Department of Molecular Neuroimmune Signaling, Shemyakin-Ovchinnikov Institute of Bioorganic Chemistry, Russian Academy of Sciences, Moscow, Russia; ^2^Faculty of Medicine, Walther Straub Institute of Pharmacology and Toxicology, Ludwig-Maximilians-Universität München, Munich, Germany; ^3^Laboratory of Cellular Neurobiology, Institute of Cell Biophysics, Russian Academy of Sciences, Pushchino, Russia

**Keywords:** nicotinic acetylcholine receptor, α9α10 subtype, *Xenopus laevis* oocytes, α–neurotoxin, α-conotoxin, granulocytes, interleukin-10, inflammation

## Abstract

Unlike most neuronal nicotinic acetylcholine receptor (nAChR) subunits, α7, α9, and α10 subunits are able to form functional homo- or heteromeric receptors without any β subunits. While the α7 subtype is widely distributed in the mammalian brain and several peripheral tissues, α9 and α9α10 nAChRs are mainly found in the cochlea and immune cells. α-Conotoxins that specifically block the α9α10 receptor showed anti-nociceptive and anti-hyperalgesic effects in animal models. Hence, this subtype is considered a drug target for analgesics. In contrast to the α9α10-selective α-conotoxins, the three-finger toxin α-bungarotoxin inhibits muscle-type and α7 nAChRs in addition to α9α10 nAChRs. However, the selectivity of α-neurotoxins at the α9α10 subtype was less intensively investigated. Here, we compared the potencies of α-conotoxins and α-neurotoxins at the human α9α10 nAChR by two-electrode voltage clamp analysis upon expression in *Xenopus* oocytes. In addition, we analyzed effects of several α9α10-selective α-conotoxins on mouse granulocytes from bone marrow to identify possible physiological functions of the α9α10 nAChR subtype in these cells. The α-conotoxin-induced IL-10 release was measured upon LPS-stimulation. We found that α-conotoxins RgIA, PeIA, and Vc1.1 enhance the IL-10 expression in granulocytes which might explain the known anti-inflammatory and associated analgesic activities of α9α10-selective α-conotoxins. Furthermore, we show that two long-chain α-neurotoxins from the cobra *Naja melanoleuca* venom that were earlier shown to bind to muscle-type and α7 nAChRs, also inhibit the α9α10 subtype at nanomolar concentrations with one of them showing a significantly slower dissociation from this receptor than α-bungarotoxin.

## Introduction

Nicotinic acetylcholine receptors (nAChRs) consisting of α9 subunits were originally discovered in the hair cells of the inner ear (Elgoyhen et al., 1994) and were found to be involved in hearing. Later, the accessory α10 subunit was identified (Elgoyhen et al., 2001) and both homomeric α9 and heteromeric α9α10 assemblies were found to form functional nAChRs receptors. The α9α10 nAChR is distinguished from other members of the nAChR family by its sensitivity to several ligands of muscarinic AChRs and agonists of other Cys-loop receptors, such as type A γ-aminobutyric acid (GABA_A_), glycine, and 5-hydroxytryptamine type 3 (5-HT_3_) receptors (Rothlin et al., 1999). Moreover, typical nAChR agonists (nicotine and epibatidine) act as antagonists at α9 (Verbitsky et al., 2000) and α9α10 receptors (Moglie et al., 2021).

α9α10 nAChRs have also been found in a number of immune cells (Peng et al., 2004; Galvis et al., 2006; Grau et al., 2019) where they have been involved in the modulation of pain signals and regulation of inflammatory processes (McIntosh et al., 2009; Grau et al., 2019). Together with a proposed role in cancer development (Sun et al., 2020a) this makes them promising targets for drug development with an emphasis on inhibitory ligands.

Well-recognized tools in nAChR research are snake venom α-neurotoxins which are classified into short-chain and long-chain ones (Barber et al., 2013). Short-chain α-neurotoxins comprising 60–62 amino acids residues and four disulfide bridges inhibit muscle-type nAChRs with high selectivity. Long-chain α-neurotoxins containing 66–75 amino acid residues and five disulfide bridges additionally block α7 nAChRs and, moreover, also inhibit α9α10 nAChRs (Elgoyhen et al., 2001; Chandna et al., 2019) and thus must be considered rather non-selective. In contrast, α-conotoxins, small neurotoxic peptides from venomous *Conus* marine mollusks, are much more selective. They not only allow to distinguish the muscle-type nAChRs from the neuronal ones, but provide markers for individual neuronal subtypes (Ellison et al., 2006; Vincler et al., 2006; Dutertre et al., 2017; Ho et al., 2020). In particular, the naturally occurring α-conotoxins Vc1.1 and RgIA as well as the αO-conotoxin GeXIVA (and their derivatives) show high affinity for α9α10 nAChRs and have been investigated in models of neuropathic pain (Luo et al., 2015; Huynh et al., 2020; Sun et al., 2020b).

At the Shemyakin-Ovchinnikov Institute of Bioorganic Chemistry in collaborations with several other laboratories, snake-venom α-neurotoxins, and peptides, as well as synthetic α-conotoxins are applied to investigate the structure and function of nAChRs, with a focus on the muscle-type and α7 nAChRs (Tsetlin, 2015; Dutertre et al., 2017; Tsetlin et al., 2021). We have recently published the synthesis of oligoarginine inhibitors of the α9α10 nAChRs (Lebedev et al., 2019), and analyzed the interaction of αO-conotoxin GeXIVA with the acetylcholine-binding protein (AChBP) and with the soluble ligand-binding domain (LBD) of the α9 subunit (Kryukova et al., 2018). In collaboration with crystallographers from Hellenic Pasteur Institute (Athens, Greece), we contributed to the determination of the X-ray structure of α-conotoxin RgIA in complex with the LBD of the α9 subunit (Zouridakis et al., 2019). We further found that α-conotoxins RgIA and Vc1.1 influence cytosolic Ca^2+^ concentration, cell adhesion, and generation of reactive oxygen species in murine bone marrow granulocytes (Safronova et al., 2021). In this special issue on the α9α10 nAChR subtype, we will briefly discuss these findings and (1) report the selectivity and potency of novel α-neurotoxins from *Naja melanoleuca* snake venom at human α9α10 nAChRs and (2) present new data showing that α9α10-selective α-conotoxins potentiate release of the anti-inflammatory cytokine interleukin-10 (IL-10) from murine granulocytes.

## Materials and Methods

### Materials

Percoll, trypan blue, lipopolysaccharide from *E. coli* O55:B5l were purchased from Sigma-Aldrich (St. Louis, United States). PE-anti-mouse Ly-6G/Ly-6C antibody, RB6-8C5 clone was from BioLegend (San-Diego, United States). DMEM, fetal bovine serum (FBS), L-glutamine, penicillin, streptomycin, amphotericin B were from Gibco (United States). Nicotine bitartrate and acetylcholine chloride (ACh) were purchased from Sigma-Aldrich (St. Louis, United States). Chemicals for oocyte buffers and electrophysiology were purchased from Carl Roth (Karlsruhe, Germany), except for BAPTA-AM [1,2-bis(o-Aminophenoxy)ethane-N,N,N′,N′-tetraacetic Acid Tetra(acetoxymethyl) Ester] which was purchased from Calbiochem (Merck KGaA, Darmstadt, Germany).

The synthesis of α-conotoxins MII, RgIA, and Vc1.1 was described in Safronova et al. (2021), GeXIVA and PeIA in Kryukova et al. (2018). α-Neurotoxins were isolated from snake venoms: long-chain Tx-NM2 and Tx-NM3-1 from *N. melanoleuca* venom (Son et al., 2021); long-chain neurotoxin I (NT I) and short-chain neurotoxin II (NT II) from *N. oxiana* and α-bungarotoxin (α-Btx) from *Bungarus multicinctus* (Kudryavtsev et al., 2015); non-conventional WTX and long-chain α-cobratoxin (α-Ctx) from *N. kaouthia* (Utkin et al., 2001; Osipov et al., 2008, respectively). Peptide neurotoxin azemiopsin (AZE) was synthesized as described (Utkin et al., 2012).

### Nicotinic Acetylcholine Receptor, cDNAs, RNA Preparation, and Oocyte Injection

The human α3 (GenBank: U62432.1), α4 (GenBank: L35901.1, with silent base exchanges to reduce GC content), β2 (GenBank: X53179.1), and β4 (GenBank: U48861.1) nAChR subunits were synthesized (FragmentGene service, Genewiz) and cloned into the pNKS2 vector (Gloor et al., 1995) by Gibson assembly. cDNAs of human α7 in pMXT and α9 and α10 in pT7TS vectors were a gift from David Adams (Illawara Health and Medical Research Institute, Wollongong University, Australia). cRNA was synthesized from linearized plasmids using the SP6 mMessageMachine kit (Invitrogen, Thermo Fisher Scientific, United States). *Xenopus laevis* females were obtained from Nasco (Fort Atkinson, WI, United States) and kept at the core facility animal models (CAM) of the biomedical center (BMC) of LMU Munich, Germany (Az:4.3.2-5682/LMU/BMC/CAM) in accordance with the EU Animal Welfare Act. To obtain oocytes, frogs were killed with an overdose of MS222. Death was confirmed by cardiac pucture/exsanguation. Oocytes were extracted and injected with 50-nl aliquots of cRNA (0.75 μg/μl, α9:α10 in 3:1 subunit ratio, all other cRNAs with 0.5 μg/μl and the indicated α:β ratios), and kept at 16°C in sterile-filtered ND96 (96 mM NaCl, 2 mM KCl, 1 mM CaCl_2_, 1 mM MgCl_2_, 5 mM HEPES, pH 7.4) containing 5 μg/ml gentamicin.

### Electrophysiological Recordings and Data Analysis

Two-electrode voltage clamp (TEVC) recordings were performed 3 days after cRNA injection at a holding potential of −70 mV. α9α10-expressing oocytes were incubated for 2–4 h in 30–100 mM BAPTA prior to recordings to obtain stable current responses. Pipettes (resistances < 1 MΩ) were pulled from borosilicate glass and filled with 3 M KCl. Membrane currents were recorded with a Turbo Tec 05X amplifier (npi electronic, Tamm, Germany), filtered at 200 Hz, and digitized at 400 Hz using CellWorks software. For α9α10 recordings, the perfusion medium was automatically switched between ND115 recording solution (115 mM NaCl, 2.5 mM KCl, 1.8 mM CaCl_2_, 10 mM HEPES, pH 7.4) with or without agonist (40 μM ACh) using a custom-made magnetic valve system as described in Giribaldi et al. (2020). Briefly, ACh pulses were applied for 2 s at 4-min intervals. After each agonist application, cells were superfused for 54 s with ND115, followed by a 3 min interval with no perfusion during which the toxin was mixed from a 10-fold stock into the static bath. Toxins were applied when responses to three consecutive agonist applications differed by less than 10%. ACh-evoked responses following toxin incubation were normalized to the ACh responses before toxin exposure. Data were analyzed with GraphPad Prism version 9 (GraphPad Prism, RRID: SCR_002798). Dose-response curves were fit to the data using the Hill equation: % response = Bottom + (Top-Bottom)/[1 + 10^((LogIC_50_-X) × Hill Slope)] and constraints of 100 and 0% for Top and Bottom, respectively. Dissociation curves were fit to the data with the equation: % response = [response (time 0) − plateau] × exp(−K × time) + plateau. Recordings for all other subtypes were performed in ND96 recording solution (96 mM NaCl, 2 mM KCl, 1 mM CaCl_2_, 1 mM MgCl_2_ 5 mM HEPES, pH 7.4) using the same protocol. BAPTA-AM was not well tolerated by the oocytes and a baseline correction was applied to compensate for baseline shifts in repetitive measurements. Recordings were denoised using a 20 Hz Gaussian lowpass filter. All measurements were performed with oocytes from at least two different frogs.

### Animals

BALB/c male mice (21–23 g of weight) were obtained from the Branch “Stolbovaya” of the Scientific Biomedical Technology Centre of the Federal Medico-Biological Agency (Moscow region, Russia). The ethical protocol No. 2019/5 based on the Manual for Working with Laboratory Animals No. 57 (30.12.2011) of the Institute of Cell Biophysics of the Russian Academy of Sciences (Pushchino, Russia) was applied for all manipulations with animals.

### Granulocyte Isolation

Polymorphonuclear neutrophilic granulocytes (PMNs) were isolated from murine bone marrow using the previously described method (Safronova et al., 2021). Shortly, a cell suspension was obtained after washing out murine femur, tibia, and humerus with cold RPMI-1640 medium and layered on a Percoll gradient (78, 62.5, and 55% in PBS). After centrifugation (1,500 × g, 35 min, 4°C), cells were collected between the 78 and 62.5% layers and washed thrice with RPMI-1640 medium. PMNs accounted for nearly 90% of the isolated cell population as estimated by expression of granulocyte maturity marker Gr-1 using the PE-anti-mouse Ly-6G/Ly-6C antibody (RB6-8C5 clone) for FACS analysis (EPICS XL-MCL, Beckman Coulter, United States). The cell survival was 98% as determined by trypan blue staining. PMNs were used in the experiment after 1 h resting at 4°C.

### Enzyme-Linked Immunosorbent Assay for IL-10

In each well of a 48-well plate, 600 μl of culture medium (DMEM, 10% FBS, 2 mM L-glutamine, 100 units/ml penicillin, 100 μg/ml streptomycin, and 250 ng/ml amphotericin B) were added. 1.2 × 10^6^ cells were added in each well and incubated for 20 min at 37°C in a CO_2_-incubator (Sanyo, Japan). After cell adhesion, LPS from *E. coli* (O55:B5, 10 ng/ml final concentration) was added or not (control) followed by 30 min incubation at 37°C. Then 100 μM nicotine or one of the α-conotoxins (200 nM MII, 10 nM RgIA, 25 nM Vc1.1, 10 nM PeIA, or 10 nM GeXIVA) were added to the LPS-treated cells and cells were incubated for 23 h. The total volume of each sample was 612 μl. All incubations were carried out in a CO_2_-incubator (5% CO_2_, 37°C, 100% humidity). Afterward, the supernatants from each well were collected into individual reaction tubes (Eppendorf, Germany) and centrifuged (2,000 × g, 10 min, 4°C). Measurement of IL-10 concentrations was carried out using a mouse IL-10 ELISA kit (ab108870, Abcam, United Kingdom) according to the manufacturer’s protocol for which the minimum detectable dose of IL-10 is typically ∼14 pg/ml. Optical density of the samples was measured with an Infinity F50 microplate photometer (Tecan, Grödig, Austria). IL-10 concentrations were calculated using the calibration curve in the range of 7–125 pg/ml obtained with the provided IL-10 standards.

### Statistical Analysis for Granulocyte Assay

Experiments were performed in duplicates on the cells from 9 to 12 animals. MATLAB software (MATHWORK INC., United States) was used for data analysis. The Kruskal-Wallis One Way Analysis of Variance on Ranks was used for multiple comparisons. Further the Mann-Whitney Rank Sum Test was applied to reveal significant differences between “LPS” and “LPS + any nAChR ligand” groups based on the fact that measurement of each sample was carried out independently. The average values and SEM were calculated for each of the experimental data.

## Results

### Testing Effects of α-Conotoxins on IL-10 Release From Mouse Granulocytes

mRNA for the α9 nAChR subunit was previously detected in BM-PMNs (St-Pierre et al., 2016) and recently confirmed by us (Safronova et al., 2021). In addition, we identified for the first time mRNA of the α10 subunit in these cells (Safronova et al., 2021). In support of a functional role of the α9 and/or α9α10 nAChRs in BM-PMNs, we showed that α-conotoxins RgIA and Vc1.1 induced Ca^2+^ transients, enhanced cell adhesiveness and decreased production of reactive oxygen species in these cells (Safronova et al., 2021). To further investigate the physiological roles of α9α10 nAChRs and a possible involvement in inflammation, we investigated in the present study the influence of the specific α9/α10 antagonists on IL-10 release by LPS-stimulated BM-PMNs, an *in vitro* model of inflammation.

**FIGURE 1:**
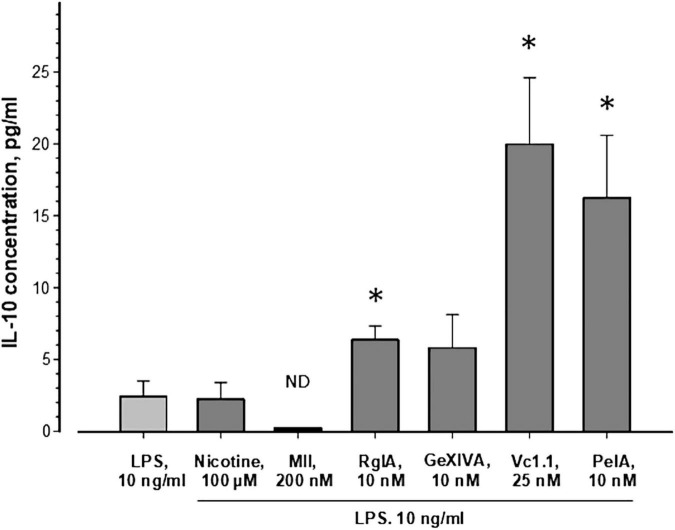
Influence of nAChR ligands on the release of IL-10 from murine bone marrow granulocytes. Cells were incubated in a medium containing 10 ng/ml lipopolysaccharide from *E. coli* without or with nicotine or α-conotoxins, as indicated. IL-10 concentrations were measured in supernatants after 23 h of cell incubation using a mouse IL-10 ELISA kit (ab108870, Abcam, United Kingdom). The average values ± SEM of 9–12 independent measurements, each performed in duplicates, are shown. The Kruskal-Wallis One Way Analysis of Variance on Ranks and the Mann-Whitney Rank Sum Test were used. ND, not detectable; *p < 0.05 compared to the cells treated with LPS only.

As seen in Figure 1, nicotine (100 μM) application in addition to LPS did not change the release of IL-10 and addition of 200 nM α-conotoxin MII (employed as a control for α3*, α6*, and α7 nAChRs) did not influence significantly the IL-10 level. These results indicate that MII-sensitive α3*, α6*, and α7 nAChR subtypes are not involved in IL-10 release. Interestingly, application of α-conotoxin RgIA (10 nM) resulted in nearly threefold increased IL-10 release, while it increased almost 6 times in the presence of α-conotoxins Vc1.1 (25 nM) or PeIA (10 nM). Application of α-conotoxin GeXIVA (10 nM) showed a tendency to increase the cytokine IL-10 release, but a statistically significant effect was not achieved. Although the minimum detectable concentration of IL-10 for the Abcam kit is typically ∼14 pg/ml, using our standard calibration curve we detected as low IL-10 concentration as 7 pg/ml. This kit was also used before for the measurement of fairly low IL-10 concentrations: 5–20 pg/ml (Khezri et al., 2019), 10–13 pg/ml (Zhang et al., 2019; Ai et al., 2020), and 10–38 pg/ml (Monga et al., 2019). It should be mentioned that the concentrations of IL-10 detected in the presence of α-conotoxins Vc1.1 and PeIA (Figure 1) exceeded the minimum detectable concentration of the Abcam kit. The concentrations for α-conotoxins RgIA, GeXIVA, PeIA, and Vc1.1 were chosen around their IC_50_ values at the α9α10 nAChR (McIntosh et al., 2005; Vincler et al., 2006; Ellison et al., 2008; Luo et al., 2015). Together, the results suggest that α9-containing nAChRs, that may be activated by endogenous ACh secreted by cells into the culture media, prevent IL-10 release.

IL-10 induces analgesic and anti-inflammatory activity (Saadane et al., 2005; da Silva et al., 2015). The increased IL-10 production in our experiments therefore provides a possible mechanism how α-conotoxins (RgIA, Vc1.1, and PeIA) *via* blockade of α9 and/or α9α10 nAChRs could exert protective effects against pain and progression of inflammation.

### Potencies of Snake Venom Neurotoxins at the Human α9α10 Nicotinic Acetylcholine Receptor

As mentioned above, α9α10 nAChRs show unusual pharmacological properties in comparison to other nAChRs and represent potential drug targets. The snake venom toxins α-Btx and α-Ctx have been shown to inhibit rat α9 nAChRs (Elgoyhen et al., 1994) and human α9α10 (Chandna et al., 2019) in addition to α7 and muscle type receptors. To further evaluate the potential of snake venom toxins as α9α10 ligands, we compared the potency and subtype selectivity of the long-chain α-neurotoxins Tx-NM2, Tx-NM3-1, NT I, the short-chain α-neurotoxin NT II, the non-conventional neurotoxin WTX, and the linear peptide AZE on the human α9α10 nAChRs expressed in *X. laevis* oocytes.

**FIGURE 2:**
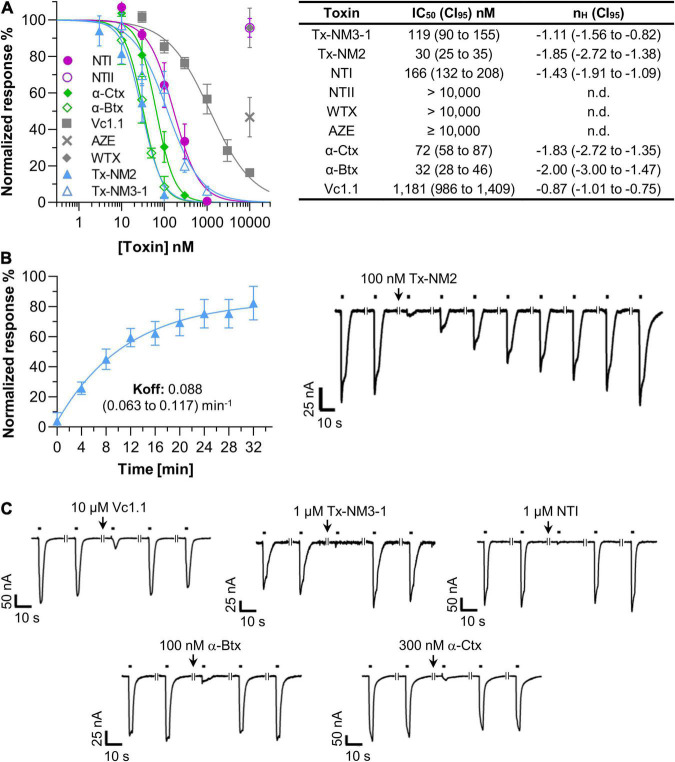
Potencies of snake toxins at the *Xenopus laevis* oocyte-expressed human α9α10 nicotinic acetylcholine receptor (nAChR). **(A)** Dose-Response curves and half-maximal inhibitory concentrations (IC_50_) values of the indicated toxins. Responses to 2 s pulses of 40 μM acetylcholine (ACh) were measured at a potential of –70 mV. Toxins were pre-incubated for 3 min in a static bath. nH: Hill-slope. 95% confidence intervals (CI_95_) are given in parenthesis. Note that the high values of the Hill coefficients suggest that a 3 min pre-incubation with the toxins is insufficient for complete binding and IC_50_ values might therefore be underestimated (compare Supplementary Figure 1). However, for practical reasons (decreasing stability of oocytes in the static bath, need of large toxin amounts in case of superfusion), all measurements were performed after 3 min pre-incubation. **(B)** Recovery of α9α10 current responses after a block induced by 100 nM Tx-NM2 Representative current traces are shown. Black bars indicate application of 40 μM ACh. Interruptions in the traces indicate a 4 min interval. **(C)** Representative current traces showing the fast dissociation of the indicated toxins from the α9α10 nAChR. Recording conditions are as in **(B)**. Each point represents the mean of 3–5 measurements from different oocytes of least two different frogs. Error bars represent the standard deviation (S.D.).

All experiments were performed with an injected α9:α10 cRNA ratio of 3:1 as this resulted in most robust current responses. To validate our recordings conditions, we first used α-conotoxin Vc1.1 as a positive control. Figure 2A shows that the ACh-activated currents were efficiently inhibited by α-conotoxin Vc1.1 with an IC_50_ value of 1.18 μM, very similar to previously described values (Yu et al., 2013, 2018).

Using the same protocol, we next determined the IC_50_ values of the snake toxins at the α9α10 nAChR subtype. Figure 2A shows that the long-chain α-neurotoxins from *N. melanoleuca* (Tx-NM2 and Tx-NM3) inhibited this receptor with potencies close to those of α-Btx (IC_50_ 32 nM) and α-Ctx (72 nM). Interestingly, the most potent toxin Tx-NM2 (IC_50_ 30 nM) needed 30 min to fully dissociate from the receptor (Figure 2B). In contrast, all other toxins tested in this study, including α-Btx and α-Ctx, allowed full recovery of the ACh responses within 4 min (Figure 2C).

A somewhat weaker potency was found for the long-chain α-neurotoxin NT I from the *N. oxiana* venom (IC_50_ 166 nM, Figure 2). In contrast, the short-chain α-neurotoxin NT II from this species failed to inhibit the α9α10 nAChR at concentrations up to 10 μM. All short-chain α-neurotoxins including NT II were previously found to lack affinity to the α7 nAChR but their possible effect at the α9α10 nAChR was not analyzed before. The non-conventional neurotoxin WTX from *Naja kaouthia*, which at micromolar concentrations binds to both the muscle-type and α7 nAChRs (Utkin et al., 2001), also did not affect α9α10 currents. AZE (Utkin et al., 2012), a linear peptide from the venom of *Azemiops feae* viper showed only a weak inhibition of about 40% at a concentration of 10 μM (Figure 2).

To estimate the nAChR subtype selectivities of the above toxins, we next measured their ability to inhibit human α7, α2β2, α3β2, α4β2, and muscle-type nAChRs at 1 μM concentration. As seen in Table 1, none of the toxins inhibited neuronal α2β2, α3β2, or α4β2 nAChR subtypes. Similar to α-Btx and α-Ctx, the toxins Tx-NM3-1, Tx-NM2, and NT-I, while being most effective against the α9α10 nAChRs, were also potent inhibitors of α7 and muscle-type receptors, indicating similar binding motives for long-chain α-neurotoxins in these subtypes. The short chain α-neurotoxin NT II and the linear peptide AZE selectively inhibited the muscle-type receptor, as previously reported (Utkin et al., 2001, 2012).

**TABLE 1:** Normalized responses of human nAChR subtypes to the indicated acetylcholine (ACh) concentration after 3 min pre-incubation with 1 μM of the indicated toxins.

					(α1)2β1εδ
	α4β2 (5:1)	α3β2 (1:1)	α2β2 (1:1)	α7	(2:1:1:1)
ACh conc.	100 μM	100 μM	100 μM	100 μM	30 μM
Tx-NM3-1	80 ± 6%	67 ± 6%	96 ± 2%	2 ± 2%**	1 ± 1%*
Tx-NM2	84 ± 9%	68 ± 7%	99 ± 2%	2 ± 2%**	1 ± 1%*
WTX	99 ± 1%	101 ± 2%	95 ± 4%	99 ± 2%	100 ± 7%
NT I	101 ± 1%	101 ± 1%	94 ± 2%	2 ± 2%**	2 ± 3%**
NT II	99 ± 2%	101 ± 1%	96 ± 3%	91 ± 4%	0 ± 0%*
AZE	100 ± 1%	101 ± 1%	98 ± 3%	101 ± 2%	**13 ± 10%**

*Three recordings were performed on different oocytes from at least two frogs. Mean values with standard deviation (S.D.) are shown. The injected mRNA ratio is given in parenthesis for each nAChR subtype.*

* Indicates a slow off-rate of the toxin, ** indicates no off-rate of toxin within 10 min. High potency is highlighted in bold.

In conclusion, although Tx-NM2 is not selective for the α9α10 nAChR, it has the highest affinity for this subtype and is the only venom-derived toxin that shows a slow dissociation from this receptor.

## Discussion

Research at the Shemyakin-Ovchinnikov Institute originally concentrated on muscle-type and α7 nAChRs but recently focused also on α9α10 subtypes and their interactions with α-conotoxins and three-finger proteins, namely α-neurotoxins and human proteins of the Ly6 family (see review Tsetlin et al., 2021).

### Structural Studies on Nicotinic Acetylcholine Receptors in Complex With Toxins

While cryo-electron microscopy structures of the *T. marmorata* nAChR (Unwin and Fujiyoshi, 2012) and the X-ray structure of the α4β2 nAChR (Morales-Perez et al., 2016) are known, the number of nAChR structures in complexes with peptide and protein neurotoxins is limited. Advances in cryo-EM only recently revealed the structures of the *Torpedo* nAChR (Rahman et al., 2020) and the human α7 nAChR in complex with α-Btx (Noviello et al., 2021). Previously, binding modes of α-neurotoxins or α-conotoxins were based on the X-ray analysis of their complexes with the AChBP, a versatile surrogate of the LBD of nicotinic and other Cys-loop receptors. Our laboratories participated in the structure determination of AChBP in complex with α-conotoxins specific for the α7 (PnIA analog and ImI), α3β2 (LvIA), and α3β4 (GIC) receptors (Celie et al., 2005; Ulens et al., 2006; Lin et al., 2016; Zhu et al., 2020). Recently the combination of alanine scanning, site-directed mutagenesis, computer modeling, and X-ray crystallography of the AChBP in complex with α-conotoxin LvIA and its synthetic analogs, identified several residues in the β2 subunit that confer LvIA specificity for the α3β2 nAChR (Zhu et al., 2020). In collaboration with Greek crystallographers, who earlier demonstrated the similarity between the α-Btx structures in complexes with AChBP and the heterologously expressed α9 LBD (Zouridakis et al., 2014), the first X-ray structure of α-conotoxin RgIA in complex with the α9 LBD was solved and, based on computer modeling, a model for RgIA binding at the α9-α10 interface was proposed (Zouridakis et al., 2019).

Most α-conotoxins bind at the orthosteric ligand binding sites in different nAChRs subtypes. Because of the high homology of such sites in all nAChR subtypes, drugs that bind at more diverse allosteric sites would have a higher chance to act in a subtype-selective way (Wang and Lindstrom, 2018). In this respect, αO-conotoxin GeXIVA with analgesic activity (Wang et al., 2019) is of interest. In TEVC experiments it inhibited the rat α9α10 nAChR at nanomolar concentrations (Luo et al., 2015) by binding exclusively to an allosteric site, thus opening up a strategy for subtype-selective targeting. However, competition with radioactive α-Btx revealed that αO-conotoxin GeXIVA also binds with a lower affinity (at micromolar concentrations) to the orthosteric sites in the monomeric α9 LBD and in the pentameric *Aplysia californica* AChBP (Kryukova et al., 2018).

### Toxins as Tools for Functional Studies

Due to their high subtype selectivity, α-conotoxins might provide a basis for the development of novel drugs. Most interesting are α-conotoxin RgIA, αO-conotoxin GeXIVA, and their derivatives, which have analgesic properties and target α9α10 nAChRs (Wang et al., 2019; Huynh et al., 2020). The anticancer activity of several nAChR subtype selective α-conotoxins was also tested (Terpinskaya et al., 2015, 2020). The application of α-conotoxins PnIA, RgIA, ArIB[V11L,V16D], or MII together with either baicalein or indomethacin to Ehrlich carcinoma enhanced the antitumor activity several-fold (Osipov et al., 2020). However, while baicalein exerted antiproliferative and cytotoxic effects also on C6 glioma cells, α-Ctx and α-conotoxin RgIA on the contrary promoted proliferation of these cells (Terpinskaya et al., 2021). Thus, further research is required to elucidate the role of nAChRs in different tumor cell lines and environments.

α-Conotoxins are not only convenient tools for structure-function studies on heterologously expressed nAChRs, but also for characterization of their physiological roles in native tissues. Here, we extended a previous study on the involvement of α9α10 nAChRs in mouse granulocyte functions and found that α-conotoxins (RgIA, Vc1.1 and PeIA) significantly increased the release of IL-10 (see Figure 1), which is known to produce analgesic and anti-inflammatory effects (Saadane et al., 2005; da Silva et al., 2015). We suggest that α9-containing nAChRs activated by endogenous ACh may prevent IL-10 release. Similarly, the inhibition of hybridoma cell proliferation by α-Ctx or WTX has been explained by prior action of endogenously released ACh (Skok et al., 2003). There is also evidence in the literature that non-neuronal ACh released by immune cells regulates immune functions *via* nAChRs (Mashimo et al., 2021) and ACh synthesis was demonstrated in granulocytes (Neumann et al., 2007). Although there are no data showing that α9α10 nAChRs in murine bone marrow granulocytes are constitutively active, we have previously shown effects of α9α10 antagonists, RgIA and Vc1.1 in the absence of agonists, on functions of murine bone marrow granulocytes (Safronova et al., 2021). Similar results were obtained by other groups for the action of different nAChR antagonists on immune cells (Razani-Boroujerdi et al., 2007; Zazueta-Favela et al., 2019). Together with previous findings (Safronova et al., 2016, 2021; Serov et al., 2021), this supports the participation of the α9 and/or α9α10 nAChR in the anti-inflammatory processes and might help to explain the analgesic action of compounds inhibiting this receptor.

### Subtype-Selectivity of Snake Toxins

It was earlier shown that α-Btx and α-Ctx can inhibit distinct subtypes of ionotropic GABA_*A*_ receptors (McCann et al., 2006; Hannan et al., 2015; Kudryavtsev et al., 2015) and similar properties were found for the recently isolated *N. melanoleuca* long-chain α-neurotoxins (Son et al., 2021). However, *N. melanoleuca* Tx-NM2, in contrast to α-Btx and α-Ctx, distinguishes the two ACh binding sites in the *Torpedo* receptor (Son et al., 2021). Here we checked if the *N. melanoleuca* toxins can also interact with the α9α10 nAChRs and whether their binding to this nAChR subtype would differ from that of α-Btx and α-Ctx.

As seen in Figure 2A, both *N. melanoleuca* toxins inhibit the α9α10 nAChRs with IC_50_ values of 30 nM (Tx-NM2) and 119 nM (Tx-NM3-1), the first one being slightly more potent than α-Btx or α-Ctx. We also tested the ability of a series of toxins from other venoms to interact with the α9α10 nAChRs. A relatively high affinity (166 nM) was detected for the NT I, a long-chain α-neurotoxin from *N. oxiana*. No activity was detected for short-chain NT II, which is not surprising since short-chain α-neurotoxins are known to bind also very weakly to the α7 nAChR. No strong inhibition was found with non-conventional toxin WTX as well. Analysis of the linear peptide AZE that does not contain disulfide bonds was interesting because it was earlier shown to inhibit the muscle-type nAChR (Utkin et al., 2012) and because other linear peptides, oligoarginines, inhibit various nAChR subtypes including the α9α10 nAChRs quite potently (Lebedev et al., 2019). However, no efficient inhibition by AZE was detected at the α9α10 nAChR (Figure 2A and Table 1).

Thus, Tx-NM2 appears most promising for α9α10 nAChR research. It has the highest affinity and dissociates significantly slower from this receptor than all other toxins tested in this study. However, Tx-NM2 was also the most active against the earlier tested nAChR and GABA_*A*_ receptor subtypes (Son et al., 2021). Nevertheless, it is the first described snake toxin that shows such high affinity at the human α9α10 receptor and provides a valuable basis to elucidate critical determinants for α9α10 selectivity and for the development of α9α10 nAChR labels.

## Data Availability Statement

The original contributions presented in the study are included in the article/Supplementary Material, further inquiries can be directed to the corresponding author/s.

## Ethics Statement

The animal study was reviewed and approved by the Commission for the Rules for the Treatment of Animals [The protocol No. 2019/5] of the Institute of Cell Biophysics of the Russian Academy of Sciences (Pushchino, Russia).

## Author Contributions

VT planned the project, wrote the first draft and together with AN, IS, YH, and YU finalized the manuscript. DK, EK, and LS contributed to the essential materials. DS, YH, PS, and VS performed the experiments. AN, YH, IK, and VS analyzed and interpreted the data. YU, VT, VS, and AN led the project. VT, YU, AN, VS, and IK contributed to the funding acquisition. All authors contributed to, reviewed and approved the manuscript.

## Conflict of Interest

The authors declare that the research was conducted in the absence of any commercial or financial relationships that could be construed as a potential conflict of interest.

## Publisher’s Note

All claims expressed in this article are solely those of the authors and do not necessarily represent those of their affiliated organizations, or those of the publisher, the editors and the reviewers. Any product that may be evaluated in this article, or claim that may be made by its manufacturer, is not guaranteed or endorsed by the publisher.
