# Supplementary material for: Interaction of α9α10 Nicotinic Receptors With Peptides and Proteins From Animal Venoms
Source: Front Cell Neurosci. 2021 Dec 23;15:765541. doi: 10.3389/fncel.2021.765541 (PMC8732759; doi:10.3389/fncel.2021.765541)
Supplement: Supplementary file 1 [file Data_Sheet_1.DOCX]

Supporting information

**Interaction of α9α10 nicotinic receptors with peptides and proteins from animal venoms**

Victor Tsetlin^1^, Yves Haufe^2^, Valentina Safronova^3^, Dmitriy Serov^3^, Pranavkumar Shadarmarshan^2^, Lina Son^1^, Irina Shelukhina^1^, Denis Kudryavtsev^1^, Elena Kryukova^1^, Igor Kasheverov^1^, Annette Nicke^2^, Yuri Utkin^1^*

^1^Department of Molecular Neuroimmune Signaling, Shemyakin-Ovchinnikov Institute of Bioorganic Chemistry, Russian Academy of Sciences, Moscow, Russia

^2^Walther Straub Institute of Pharmacology and Toxicology, Faculty of Medicine, LMU Munich, Munich, Germany

^3^Institute of Cell Biophysics, Russian Academy of Sciences, Pushchino, Russia.

*** Correspondence:**

Yuri Utkin
utkin@ibch.ru; yutkin@yandex.ru

**

**

**Figure S1**: **Influence of antagonist incubation time on Hill slopes of dose inhibition response curves**. Human α9α10 nicotinic acetylcholine receptor (nAChR) was expressed in *Xenopus laevis* oocytes. Oocytes were clamped at -70 mV and activated by 2 s pulses of 40 µM acetylcholine (ACh). Estimated effect of a longer pre-incubation time on the dose inhibition curves of Tx-NM2 and NTI. The solid lines represent dose inhibition curves obtained with a three min pre-incubation time (as presented in Fig. 2A). The dashed lines show a trend towards lower IC_50_ and Hill slope values with longer (five min) pre-incubation time. Note that only two concentrations per toxin were measured. It can be expected that even longer preincubation times than five min would be required to reach equilibrium binding at low toxin concentrations and the effect of longer preincubation time increases with lower toxin concentrations and would result in a Hill coefficient closer to -1. Mean values with standard deviation are shown. Estimated IC_50_ values/ Hill slopes for 5 min pre-incubation of Tx-NM2 and NTI are 15 nM/-1.52, and 83 nM/-1.26, respectively. Note that that the estimated IC_50_ values are in the same order of magnitude for both toxins. n = 3-5 different oocytes for each concentration.
